# Robotic hemi-colectomy for ascending colon cancer in a patient with situs inversus totalis

**DOI:** 10.1186/s40792-024-01982-y

**Published:** 2024-08-01

**Authors:** Junki Kato, Takahisa Hirokawa, Kenji Kobayashi, Moritsugu Tanaka, Masahiro Kimura

**Affiliations:** Division of Gastroenterological Surgery, Kariya Toyota General Hospital, 5-15 Sumiyoshi-Cho, Kariya, Aichi 448-8505 Japan

**Keywords:** Colon cancer, Ascending colon, Situs inversus totalis, Robot-assisted surgery, Hemi-colectomy

## Abstract

**Background:**

Situs inversus totalis (SIT) is a rare congenital anomaly in which the thoracic and abdominal cavity structures are completely opposite to normal. Performing robot-assisted surgery in these patients is difficult because of these anomalies. A few reports have described robot-assisted surgery for rectal cancer in patients with SIT, but no reports to date have described robot-assisted surgery for colon cancer.

**Case presentation:**

A 74-year-old female presented with abdominal pain and was diagnosed with ascending colon cancer and SIT. We carefully planned the surgical procedure and performed robot-assisted hemi-colectomy. Although we used unusual port placement, the operation was performed safely. The patient was discharged without any complications.

**Conclusions:**

Robot-assisted surgery is safe and efficient for patients with anatomical anomalies.

## Introduction

Situs inversus totalis (SIT) is a rare congenital anomaly with an incidence of in 1 of 5000–10000 people [1]. The structures of the thoracic and abdominal cavities in patients with SIT are completely opposite to those in normal patients. Therefore, the surgical procedures are considered more difficult in patients with SIT because the positions of the organs and vessels are more complex. While some reports have described laparoscopic colectomy in patients with SIT, cholecystectomy, gastrectomy, and anterior resection have only been described using robotic surgery. To our knowledge, this is the first case report of robotic surgery for ascending colon cancer associated with SIT.

## Case presentation

**Fig. 1:**
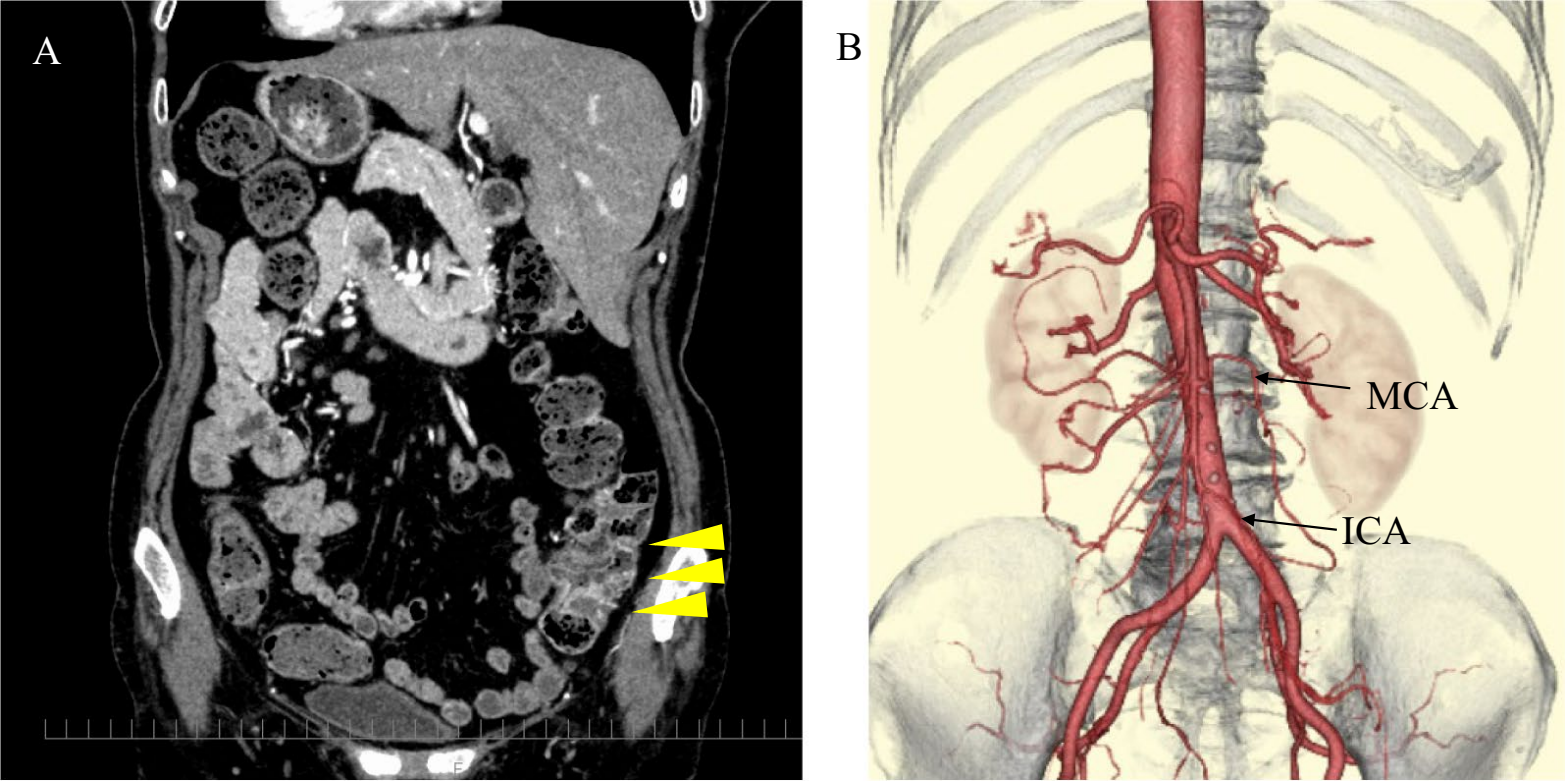
Abdominal CT and 3D-CT angiography. **A** CT revealed SIT and ascending colon cancer (yellow triangle) but no lymph node metastasis or distant metastasis. **B** CT angiography showed the ileocecal and middle colic artery. No anomalies were noted

A 74-year-old female was admitted to our hospital with abdominal pain and was diagnosed with ischemic enteritis. Her medical history included endometrial cancer with no recurrence. Colonoscopy for following enteritis was performed and revealed a polyp in the ascending colon. A pathological examination confirmed the presence of colonic adenocarcinoma. Computed tomography (CT) revealed SIT, but no lymph node or distant metastasis (Fig. 1). Based on colonoscopy observations, the cancer was subjected to endoscopic excision and endoscopic submucosal dissection (ESD) were performed. Histological examination revealed massive submucosal invasion. The cancer with over 1000 μm invasion from muscularis mucosae was recommended for surgical treatment by Japanese Society for Cancer of the Colon and Rectum (JSCCR) guidelines [2]. The patient attended our department for surgical intervention.

Three-dimensional CT angiography revealed that the structures of the thoracic cavity and all the abdominal organs were inverted from normal. The CT angiography revealed an ileocecal artery and a middle colic artery with no anomalies (Fig. 1).

**Fig. 2:**
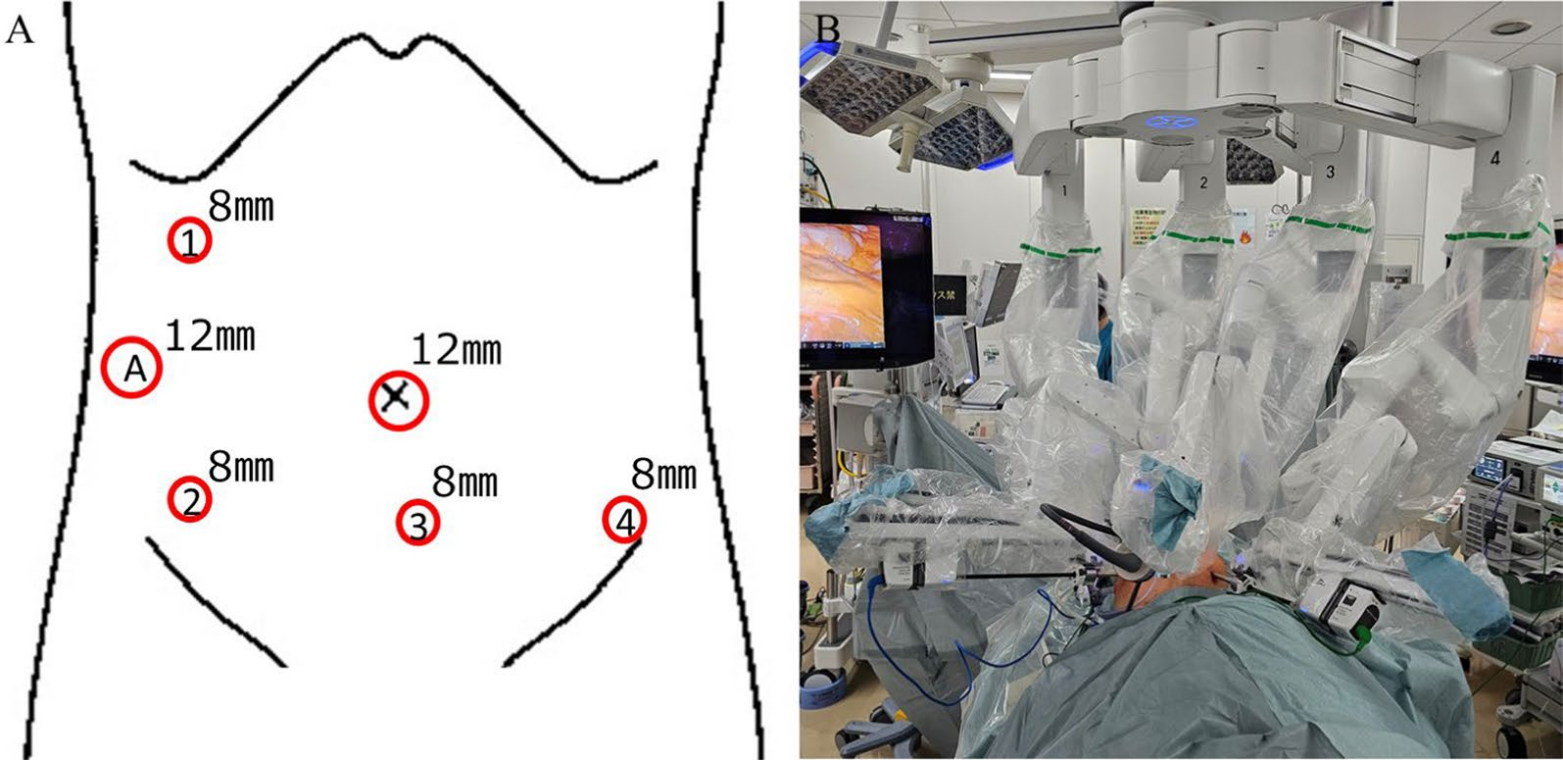
Trocar positions and arm position settings of the da Vinci® Xi. **A** We used four 8 mm robotic trocars and two 12 mm laparoscopic trocars. Assistant trocar set in right flank region. **B** Da Vinci® Xi was rolled in from the left-upper side of the patient. Forceps were placed in the first, second, and fourth arms. The endoscope was placed in the third arm

**Fig. 3:**
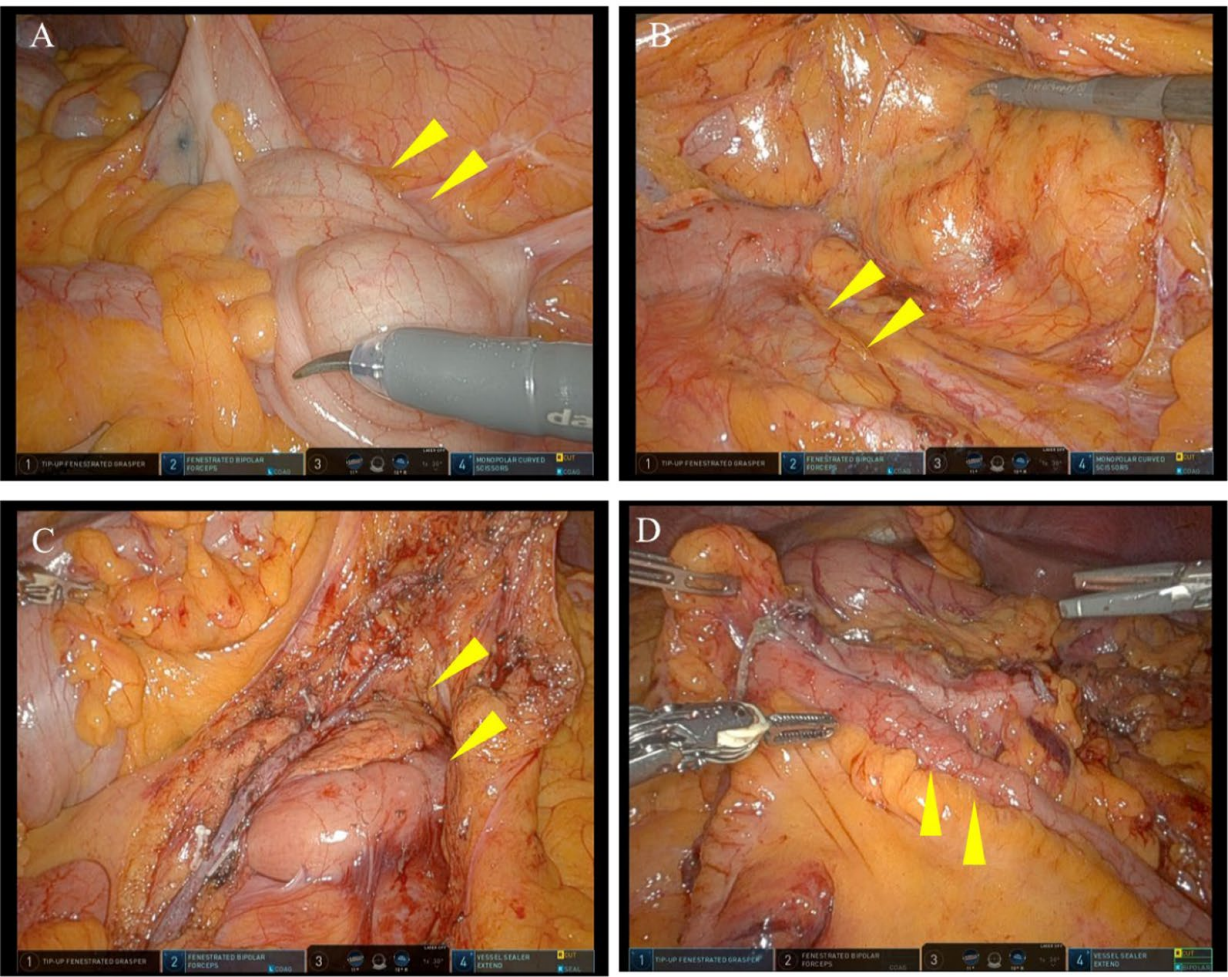
Surgical findings. **A** The ascending colon (yellow triangle) was determined to be in the left abdominal position. **B** After performing a retroperitoneal approach. Inferior vena cava (yellow triangle) runs left of aorta and duodenum runs left to right. **C** Lympadenectomy of the surgical trunk. The duodenum and pancreas (yellow triangle) were determined to be in the middle to left upper abdominal position. **D** Finished reconstruction. We performed overlapping anastomosis between the small intestine and the transverse colon (yellow triangle)

Based on these findings, a robot-assisted hemi-colectomy with radical lymphadenectomy was performed. The patients were placed in the supine position. The trocars were placed in the mirror image of a normal robotic right hemi-colectomy setup. First, a 12 mm umbilical trocar was inserted. Three robotic trocars were placed in the lower abdomen and another robotic trocar was placed in the right upper abdomen. An additional assistant 12 mm trocar placed on the right flank (Fig. 2). The patient’s head was positioned 10° downward and 10° right downward. The robot used in our hospital is the da Vinci® Xi surgical robot system. The robot was rolled in from the left side of the patient, and its arm positions are shown in Fig. 2. During the robotic procedure, we used a 30° endoscope (third arm), monopolar curved scissors (fourth arm), tip-up fenestrated grasper (first arm), and fenestrated bipolar forceps (second arm). The first and second arms were swapped to allow for surgical expansion. The surgeon controlled monopolar curved scissors (fourth arm) with the right hand and fenestrated bipolar forceps (second arm) and tip-up fenestrated grasper (first arm) on the left. Hemi-colectomy for ascending colon was performed using a retroperitoneal approach with initial peritoneal dissection between the mesocolon and retroperitoneum. A D3 lymph node dissection was performed. After resecting the colon on both sides, the proximal and distal colon were reconstructed intracorporeally using an overlapping anastomosis (Fig. 3). The operation time was 218 min, the console time was 168 min, and the estimated blood loss was 5 ml. The patient was discharged on the sixth postoperative day without complications.

## Discussion

SIT is a rare congenital anomaly in which the thoracic and abdominal cavity structures are completely opposite to normal. SIT has a reported incidence of 1 in 5000–10000 and patients may have a high risk of cancer [1, 3].

Surgery in patients with SIT is considered more difficult because of the mirror-image anatomy. To perform surgery safely, careful assessment of the anatomy using preoperative imaging is important. 3D-CT and CT angiography are useful for clarifying the anatomy [3].

Colectomy in patients with SIT is a simple change from the usual procedure and reduces the risk of misleading and damaging lesions.

Some reports on laparoscopic procedures in patients with SIT have described the surgeons’ positions, port placement, and use of devices with the nondominant hand [4–6]. Most reports suggest changing the positions of the surgeon and assistant, as well as using the same port placement. Oms and Badia [6] suggested a potential advantage for left-handed surgeons in laparoscopic surgery of patients with SIT. Right-handed surgeons encounter technical difficulties when using an energy device with their left hand during laparoscopic surgery. However, these limitations can be overcome by robot-assisted surgery (RS). In RS, multi-joint forceps allow surgeons to operate flexibly and use scissors or energy devices with their dominant hands. These advantages of RS lead to improved surgical safety and enable complicated and precise surgery for any anatomical anomaly.

There have been only four reports to date of RS for colorectal cancer in patients with SIT, and all these reports described rectal cancer (Table 1) [7–10]. This is the first report of RS for colon cancer.

**Table 1:** Reports of robotic-assisted surgery for colorectal cancer in patients with situs inversus totalis

Author	Year	Location	Complications	Blood loss	Operating time	Feature of the surgery
Leong et al. [5]	2012	Rectum	None	–	–	Square port position
Foo et al. [6]	2015	Rectum	None	100	204	Mirror port positions
Cui et al. [7]	2018	Rectum	None	50	210	Use of transanal natural orifice specimen extraction
Kasai et al. [8]	2020	Rectum	None	Minimal	194	Needed dual docking
Our case	2024	Ascending colon	None	5	218	Mirror port positions

All these studies used a da Vinci® surgical system (Xi or S). The port positions and procedures were different in each report. One report set the port positions on a horizontal line and used a retraction arm with the left hand. Other reports set port position squares and used a retraction arm with the right hand. No complications occurred in any of the patients. In the present case, the trocar positions were changed to mirror the usual locations during right hemi-colectomy. The da Vinci® Xi system was rolled in from the left side of the patient and performed with single docking. The camera was positioned as usual. The retraction arm was used with the left hand. In our procedure, anatomical understanding was straightforward because the surgical view only required a mirror image to the horizontal, and the surgeons could use forceps with coaxial camera vision. The role of the retraction arm is to retract the surgical field roughly, and there were no difficulties in controlling the retraction arm with the left hand. As a result, the creative procedure was safe.

In conclusion, we safely and efficiently performed robotic colectomy for ascending colon cancer in a patient with SIT. Our RS procedure will help surgeons perform robotic colectomy in patients with SIT.

## Data Availability

Not applicable.

## Abbreviations

SIT: Situs inversus totalis
CT: Computed tomography
ESD: Endoscopic submucosal dissection
JSCCR: Japanese Society for Cancer of the Colon and Rectum
RS: Robot-assisted surgery
